# The American and Colonial Journals: Surgery

**Published:** 1842-10

**Authors:** 


### SURGERY.

### *Cancer of the Penis.* By Dr. RIDLEY.

A gentleman, 85 years of age, who had always been healthy, but "whose habits had been for many years occasionally dissipated," complained, in 1840, of itching on the prepuce and glans penis, which were supposed to indicate lithiasis. These symptoms, however, disappeared; but about the same time a wart-like pimple, the size of a pea, and hard and red, was perceived about the insertion of the frenum into the glans. Little attention was paid to it, and it remained without any alteration for six months, when it began to extend itself. By and by, it implicated the whole glans. When the author was consulted the glans had been nearly eroded; the whole penis was involved in inflammation; the cellular tissue tumified; while about twelve lines from the scrotum was a sort of pap, with jagged edges, from which urine dribbled when the patient endeavoured to pass his water. The pain was unintermitting and excruciating. Amputation was proposed as the only remedy; but while the patient hesitated about giving his consent to this measure, suspicious tumours, filled with atheromatous or cheese-like matter, appeared in either groin. Subsequently one appeared on the pubis. The patient sank. There is no account of any post-mortem examination, and the whole case is vaguely reported.

*Medical Examiner,* [*Philadelphia, U. S.*] No. xliii. *Oct.* 23, 1841.

---

### *Iodine in Erysipelas.* By Dr. BURNS.

THE author, after having tried the usual remedies fruitlessly, had recourse to the tincture of iodine (as originally prepared by Mr. Davies of Hertford), which he employed at first diluted with rectified spirit, in the proportions of ℨj of the tincture to ℨij of the spirit. Finding good effects from the application he then applied the pure tincture, pencilling the affected part with a camel-hair brush. The effects described are, subsidence of the tumefaction, corrugation of the epidermis, disappearance of the effusion in the loose cellular substance, and, in two days, desquamation, leaving a sound unbroken surface. The author describes it as surpassing any other remedy he had ever tried.

*Medical Examiner.* No. xlv. *Nov.* 6, 1841.

